# Mitochondrial Bioenergetic, Photobiomodulation and Trigeminal Branches Nerve Damage, What’s the Connection? A Review

**DOI:** 10.3390/ijms22094347

**Published:** 2021-04-21

**Authors:** Silvia Ravera, Esteban Colombo, Claudio Pasquale, Stefano Benedicenti, Luca Solimei, Antonio Signore, Andrea Amaroli

**Affiliations:** 1 Department of Experimental Medicine, University of Genoa, 16132 Genoa, Italy; silvia.ravera@unige.it; 2 Department of Surgical and Diagnostic Sciences, University of Genoa, 16132 Genoa, Italy; esteban.colombo92@gmail.com (E.C.); clodent@gmail.com (C.P.); stefano.benedicenti@unige.it (S.B.); lucasolimei@hotmail.it (L.S.); dr.signore@icloud.com (A.S.); 3 Department of Therapeutic Dentistry, Faculty of Dentistry, First Moscow State Medical University (Sechenov University), 119991 Moscow, Russia; 4 Department of Orthopaedic Dentistry, Faculty of Dentistry, First Moscow State Medical University (Sechenov University), 119991 Moscow, Russia

**Keywords:** nerve regeneration, nerve injury, trigeminus, inferior alveolar nerve, lingual nerve, mental nerve, neuropathic pain, bioenergetic metabolism, low-level laser therapy, phototherapy

## Abstract

Background: Injury of the trigeminal nerve in oral and maxillofacial surgery can occur. Schwann cell mitochondria are regulators in the development, maintenance and regeneration of peripheral nerve axons. Evidence shows that after the nerve injury, mitochondrial bioenergetic dysfunction occurs and is associated with pain, neuropathy and nerve regeneration deficit. A challenge for research is to individuate new therapies able to normalise mitochondrial and energetic metabolism to aid nerve recovery after damage. Photobiomodulation therapy can be an interesting candidate, because it is a technique involving cell manipulation through the photonic energy of a non-ionising light source (visible and NIR light), which produces a nonthermal therapeutic effect on the stressed tissue. Methods: The review was based on the following questions: (1) Can photo-biomodulation by red and NIR light affect mitochondrial bioenergetics? (2) Can photobiomodulation support damage to the trigeminal nerve branches? (preclinical and clinical studies), and, if yes, (3) What is the best photobiomodulatory therapy for the recovery of the trigeminal nerve branches? The papers were searched using the PubMed, Scopus and Cochrane databases. This review followed the ARRIVE-2.0, PRISMA and Cochrane RoB-2 guidelines. Results and conclusions: The reliability of photobiomodulatory event strongly bases on biological and physical-chemical evidence. Its principal player is the mitochondrion, whether its cytochromes are directly involved as a photoacceptor or indirectly through a vibrational and energetic variation of bound water: water as the photoacceptor. The 808-nm and 100 J/cm^2^ (0.07 W; 2.5 W/cm^2^; pulsed 50 Hz; 27 J per point; 80 s) on rats and 800-nm and 0.2 W/cm^2^ (0.2 W; 12 J/cm^2^; 12 J per point; 60 s, CW) on humans resulted as trustworthy therapies, which could be supported by extensive studies.

## 1. Introduction

### 1.1. Trigeminal Nerve Damage

The trigeminal nerve and its branches are responsible for the sensory perception and motor function of the mandibular and maxillary area [1]. Unfortunately, injury of the trigeminal nerve in oral and maxillofacial surgery can occur, particularly at the two branches of the inferior alveolar nerve (IAN) and the lingual nerve (LN) [2].

The aetiology of nerve injuries in the mandibular area is different and can happen due to local anaesthetic injection, third molar removal, orthognathic surgery, maxillofacial trauma, pre-prosthetic surgery, endodontic treatment, salivary gland surgery, ablative surgery and cosmetic facial surgery [3]. Reliable statistics about the frequency of nerve injuries are hard to obtain since most procedures are done in a private environment, without the possibility of database compilation. Most of the current information is from group surveys, reports and retrospective data. The majority of permanent injuries result from maxillofacial surgery, such as genioplasty (cases of permanent neurosensory dysfunction after surgeries: from 3.33 to 10% of cases), sagittal split ramus osteotomy (66.6% of the surgeries) and mandibular vestibulopathy (from 50 to 100% of the surgeries); other major causes of injuries are mandible fracture (38.8%) and mandibular distraction osteogenesis (<5.0%). Other injuries are related to dental implants (from 0% to 15%), third molar removal (from 0.001% to 0.04%) and local anaesthetic injection (0.54%) [4].

When trauma occurs in the tissue, neurologic lesions are seen, such as neurapraxia (the least severe), characterised by functional and temporary interruption of nervous conduction, axonotmesis, occurring when the continuity of the axons, but not the epineural sheath, is disrupted, and neurotmesis (the most severe), involving a complete loss of nerve continuity due to fractures or iatrogenic transaction [5].

Most cases of nerve injury recover spontaneously over a varying time range [6]. Regeneration of a peripheral nerve can start almost immediately after the injury. Nerve healing usually occurs in two phases: degeneration and regeneration. Degeneration can happen by two mechanisms: segmental demyelination, which slows conduction and prevents some nerve impulses, causing alterations in sensory perceptions, or Wallerian degeneration, which occurs before nerve regeneration, described as a clearing process that prepares the distal stump for reinnervation [7].

However, despite the ability of neurapraxia and axonotmesis to recover spontaneously and the support of cortisone, or anti-inflammatory or neurotrophic drugs, the symptoms experienced by the patient do not always resolve quickly. This process often requires treatment with drugs, such as steroids, for very long periods, with the risk of developing other pathologies as a result of lowering the immune defences [8].

Conversely, neurotmesis requires reconstructive microsurgery with autologous nerve grafting to recreate the nervous continuity, but the results are still not certain, and the treatment is indicated only in certain cases.

### 1.2. Mitochondrial Dysfunction in Nerve Damage

Mitochondria display essential functions necessary to sustain and maintain cellular physiological processes [9]. Therefore, their dysfunction often leads to pathological conditions [10]. For example, the literature reports that painful neuropathies are associated with altered mitochondria [11], such as in chemotherapy-induced neuropathy [12,13], diabetic neuropathy [14], HIV-associated neuropathy [15] and Charcot-Marie-Tooth neuropathy [16], suggesting that mitochondria are mechanistically involved in these diseases.

The principal role of mitochondria is energy production through the oxidative phosphorylation machinery, composed of the four respiratory complexes and the F_o_-F_1_ adenosine triphosphate (ATP) synthase [17]. The nervous system (NS) has a high energy demand, principally used to restore the ionic balance after the generation and transmission of nerve impulses through the activity of the Na^+^/K^+^ ATPase, an ATP-dependent pump [18]. Thus, the decrease in ATP production due to alteration of mitochondrial metabolism reduces the Na^+^/K^+^ ATPase activity, making the neuron unable to restore the correct ionic equilibrium and contributing to the neuropathic pain [11,19]. 

Several authors have observed that nerve injuries led to an increase in oxygen consumption, which is not associated with an increase in ATP production [11]. This phenomenon may depend on two events: (i) an uncoupling of respiration and energy production [20] or (ii) a reversal of ATP synthase activity, resulting in ATP dissipation [21]. In each case, the reduction of ATP availability in nerve injury results in cell death and neurodegeneration, as the low level of ATP leads to neuronal depolarization, favouring neurotransmitter release, impairing their ATP-dependent reuptake [22] and increasing nociceptor excitability [11,23].

Moreover, the aerobic metabolism dysfunction correlates with an increase in reactive oxygen species (ROS) production [24], which is exacerbated by the inflammation associated with neuronal damage [25]. This creates a vicious circle, in which the alteration of respiratory complex activity increases oxidative stress, which, in turn, leads to damage of the inner mitochondrial membrane housing OxPhos machinery [26].

The altered aerobic ATP production induces a metabolic switch to anaerobic glycolysis in an attempt to restore the intracellular level of ATP [11]. However, since the ATP yield of anaerobic metabolism is 18-fold lower than the amount produced by aerobic metabolism, this results in lactic acidosis, which exacerbates the ongoing pain experienced in neuropathies [27].

Since mitochondria play a pivotal role, together with the endoplasmic reticulum (ER), in the maintenance of cytosolic calcium (Ca^2+^) levels [28], the alteration of mitochondrial metabolism, organisation and interaction with the ER leads to a cytosolic Ca^2+^ imbalance, which is considered one of the principal factors in the pathogenesis of neuropathic pain [29]. 

In each case, the reduction of ATP availability in stress conditions, such as a nerve injury, results in cell death and neurodegeneration, as the ATP reduction leads to neuronal depolarization, favouring the release of neurotransmitters and impairing their ATP-dependent reuptake [22].

### 1.3. Questions

On the basis described above, considering the role of mitochondrial bioenergetic dysfunction in the pain associated with neuropathy and in nerve regeneration, a challenge for research is to individuate new therapies able to normalise mitochondrial and energetic metabolism in order to aid nerve recovery after damage. 

In the last decade, the ability of red and near-infrared (NIR) light to interact with cells, producing a restorative effect, has been increasingly widely discussed. Low-level laser therapy (LLLT) or, more properly, photobiomodulation (PBM) is a technique involving cell manipulation through the photonic energy of a non-ionising light source (visible and NIR light), which produces a nonthermal therapeutic effect on the stressed tissue. 

Despite the increasing evidence of the potential of this therapy [30,31,32], its application in the medical field currently has detractors. In our review, we will first illustrate the reliability of the light–cell interaction underlying PBM. Secondly, according to the ARRIVE-2.0, PRISMA and Cochrane RoB-2 guidelines, we will show its applicability in preclinical and clinical studies in subjects affected by damage to the trigeminal nerve branches.

This review was based on the following questions: (1) Can photobiomodulation by red and NIR light affect mitochondrial bioenergetics? (2) Can photobiomodulation support damage to the trigeminal nerve branches? (pre-clinical and clinical studies), and, if yes, (3) What is the best photobiomodulatory therapy for the recovery of the trigeminal nerve branches?

## 2. Methods for Articles Selection

The papers concerning the red and NIR light interaction with mitochondria were independently searched by two authors (A.A. and S.R.) using the PubMed, Scopus and Cochrane databases using the following keywords: “laser phototherapy” OR “low-level laser therapy” OR “photobiomodulation” AND mitochondria OR mitochondrial OR respiratory chain. Other articles were selected from references, books or reviews. The papers were screened in compliance with the narrative review needs.

The review of preclinical (animal models) and clinical (human) data was carried out using the PRISMA guidelines (Supplemental Material, Figure S1). Papers were independently searched by four authors (A.A., E.C., C.P and S.R.) using the PubMed, Scopus and Cochrane databases. The following keywords were applied to meet the strategy of the investigation: “laser phototherapy” OR “low-level laser therapy” OR “photobiomodulation” AND “trigeminal nerve” OR “trigeminus” OR “alveolar nerve” OR “buccal nerve” OR “lingual nerve” OR “mental nerve” OR “facial nerve”. Additional studies were also identified from the references of the articles found. Authors screened the works in advance using the titles and abstracts, according to the inclusion and exclusion criteria given below. Inclusion criteria were: (1) studies published in English in journals with a peer-review process before publication, (2) original or brief articles, (3) preclinical (animal model) or clinical studies, (4) studies that aligned with the topic of the review, (5) therapies traceable to PBM and (6) clear description of the type of light-emitting diode (LED) or laser devices and treatment parameters employed. Exclusion criteria included: (1) in vitro studies; (2) LED or laser therapies not adhering to the principles of PBM; (3) studies not focused on the topic of the review and (4) types of articles, such as reviews, abstracts to congress and patents. The selection process is available in Supplementary Figure S1. Additionally, the review on preclinical research followed the ARRIVE-2.0 guidelines (Animals in Research: Reporting in Vivo Experiments) (Supplementary Table S1). The distribution of the available clinical studies was, instead, made according to their hierarchy, as shown in Supplementary Figure S2. The risk-of-bias of randomised clinical studies was evaluated according to the Cochrane RoB 2 tool. For this tool, we considered the diagnostic use of only quality scales and resulting scores as “other bias” (point F, Supplementary Table S2), which, in accordance with Higgins et al. [33], are debated ways to appraise clinical due to the risk of bias.

## 3. Result of Articles Screening

### 3.1. Mitochondria and PBM

**Figure 1:**
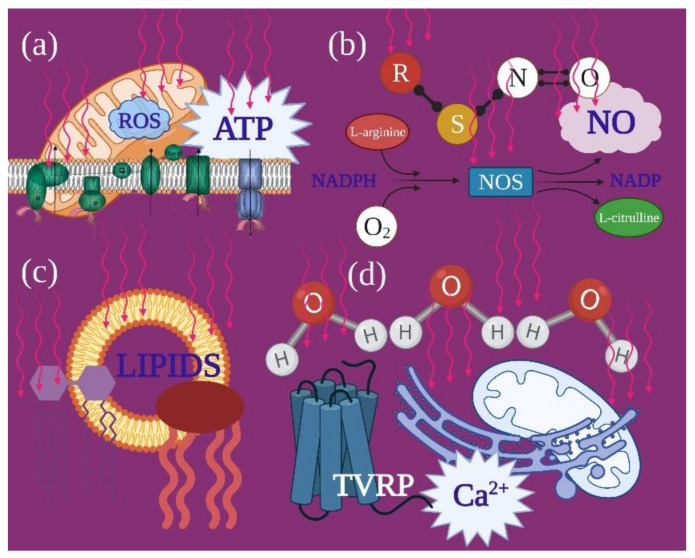
From a cellular point of view, photobiomodulation event is the results of cellular interaction sequences that from the primary target, represented by photoacceptor, move through second messengers and conclude with a modulation of the tissue’s homeostasis. The primary target can be identified in the cytochromes of the mitochondrial respiratory chain (**a**), the nitrosothiol compounds (**b**), the lipids (**c**) and the bounded water (**d**), which after the interaction with red and, particularly, infrared light, modify their energetic and vibrational state, supporting the release of ATP and ROS (**a**) and nitric oxide (**b**), as well as calcium, through the opening of voltage-dependent receptors and release from intra-organellar-sequestered reserves (**c**,**d**). Red arrows represent the laser light. Image created with BioRender.com.

The results of the review process are examined in detail in Section 4. Briefly, red and NIR wavelength lights are able to influence the bioenergetic characteristics of mitochondria by interacting with photoacceptors such as cytochromes, water, lipids, S-nitrosylated nitric oxide (NO) and transient receptor potential channels (TRPC) for Ca^2+^ through various pathways. The final results of the direct or indirect communication between light and mitochondria are the modulation of ATP and ROS production, NO release and Ca^2+^ homeostasis (Figure 1). The mechanism of action varies according to the wavelength, which may positively or negatively interfere with the resonances of photoacceptors, depending on their spatial period, influencing their conformation, properties and activities.

### 3.2. In Vivo Preclinical Studies (Animal Model, Trigeminal Branches Nerves and PBM)

Among the 13 articles selected [34,35,36] (Supplementary Figure S1 and Table S1) using the PRISMA guidelines, none completely comply with the ARRIVE Essential 10, which constitute the recommended guidelines for describing the research context. These studies fail in the accurate inclusion and exclusion criteria. Additionally, only two works [34,36] described a clear sample size calculation, while in the other articles [35,37,38,39,40,41,42,43,44,45,46], the authors only stated that “efforts were made to minimise the number of animals used and their suffering in accordance with ethical guidelines for investigations of experimental pain in conscious animals”. All of the experimental setups were approved by the relevant Regional Ethics Committee. Most studies were carried out on rats and one involved rabbits.

**Figure 2:**
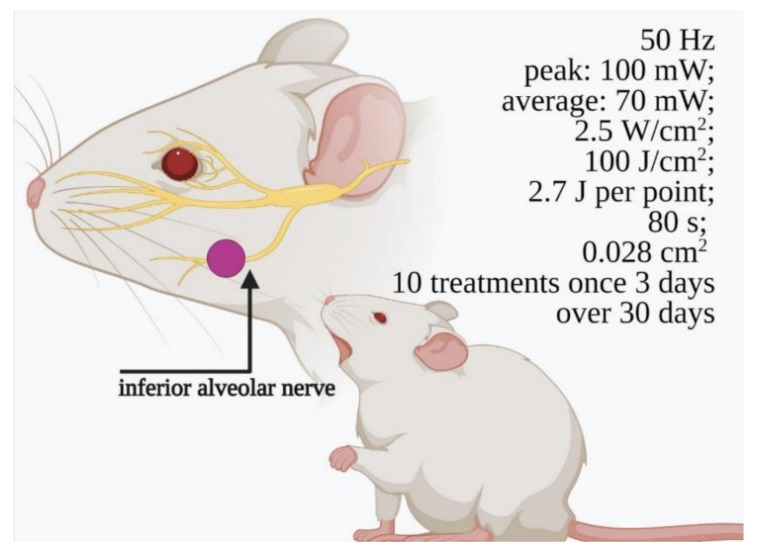
Design of the irradiation, site of irradiation and parameters, performed by Diker et al., [36] on a rat affected by an inferior alveolar nerve crush injury. Image created with BioRender.com.

Unfortunately, many articles [34,35,38,40,42,43,44,45,46] showed a high risk of bias due to a lack of randomisation and/or blinding of the procedure and were excluded (Supplementary Tables S1 and S2). Therefore, four articles [36,37,39,41] (Table 1) were selected, and the work of Diker et al. [36] was given a higher score using the ARRIVE guidelines (Supplementary Table S1). The results are summarised in Table 1 and covered more thoroughly in Section 4. Briefly, all of the papers pointed out the efficacy of PBM in treating damage to the trigeminal nerve branches. Diker et al. [36] conducted a histopathological investigation in 36 12-week-old Wistar rats (female), showing that PBM irradiation with 660 nm wavelength light did not reverse the morphological degenerative changes that occurred after IAN injury. Conversely, the 808-nm wavelength light irradiation, illustrated in Figure 2, reduced the collagen fibre deposition and oedema and preserved the unmyelinated and myelinated nerve structure fibres. Hakimiha et al. [37] observed in 72 Wistar rats (male, weighing 250–300 g) that PBM with 810 and 980-nm wavelength light improved the neurosensory recovery after IAN crush injury. However, only the application of 810-nm light had a positive effect on immunological markers compared to irradiation with 980-nm light. Sasaki et al. [41] indicated a positive effect of PBM irradiation (using a constant dose or gradual dose) on mental nerve regeneration after injury by compression lesions in 24 Wistar rats (male, weighing 250–300 g) plus 24 controls. Lastly, five New Zealand white adult rabbits (female), injured in their IAN, experienced a better recovery of axonal density, but not of axonal diameter, after PBM irradiation, compared to the samples treated with an expanded polytetrafluoroethylene graft alone [39].

### 3.3. Clinical Studies (Patient, Trigeminal Branches Nerves and PBM)

PRISMA guidelines screening (Supplementary Figure S1) resulted in 16 articles (Table 2 and Table 3) [47,48,49,50,51,52,53,54,55,56,57,58,59,60,61,62] suitable for the review, covering a total of 459 patients. Two were case reports, one was a retrospective study of clinical cases: one was a prospective study, six were case series studies and six were RCT (five double-blind and one triple-blind). According to the hierarchy available for clinical studies previously described (Supplementary Figure S2) in the Materials and Methods section, the papers ranking low the RCT (Table 3) were used to support the discussion but not considered to comply with the review questions. Therefore, the six RCT papers were judged using the Cochrane RoB 2 tool, and the results of the risk-of-bias assessment are shown in Supplementary Table S3. With the exception of one study [47], the work was carried out using scales and the resulting scores, tending to combine assessments of aspects and assign weights to different items in ways that are difficult to justify, resulting in a risk of bias. Another limitation of the papers is the lack of blinding of personnel performing the irradiation. Additionally, two papers [63,64] showed a high risk of bias due to an unclear randomisation process and were therefore excluded. Conversely, only some concerns were raised for five works and one had a low risk of bias; these original articles were considered reliable for the review process.

The results are summarised in Table 2 and Table 3 and covered more thoroughly in Section 4. Briefly, none of the patients investigated in the papers experienced dangerous effects due to PBM. Conversely, the authors [47,48,49,51,52] showed the positive effect of PBM on neurosensory recovery and, indirectly, on nerve regeneration, at various wavelengths (780, 810, 830 and 980 nm). The parameters, therapies and follow-up data are described in Table 2. However, in the study by Miloro and Criddle [50], PBM using 830-nm light did not affect IAN and lingual nerve (LN) recovery. 

In the study scored more highly using Cochrane RoB 2 [47], PBM was shown to be effective in patients requiring orthognathic surgery by Le Fort 1 (for infraorbital nerve (ION)) and bilateral sagittal split osteotomy (for IAN). This study included both women and men, aged 18–30 years. 

**Figure 3:**
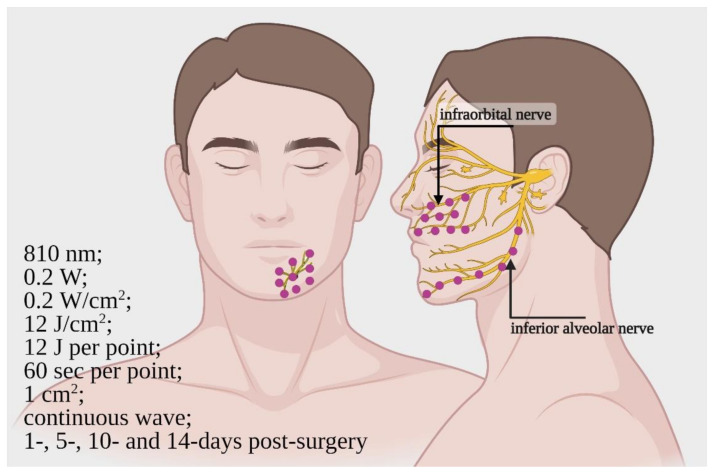
Design of the irradiation, site of irradiation and parameters, performed by Haghighat et al. [47], on patients affected by inferior alveolar nerve and infraorbital nerve damages after bilateral sagittal split osteotomy and Le Fort 1 osteotomy. Image created with BioRender.com.

The patients underwent PBM at 1, 5, 10 and 14 days post-surgery. The parameters of the PBM therapy were 810 nm; 0.2 W; 0.2 W/cm^2^; 12 J/cm^2^; 12 J; 60 s per point; 1 cm^2^; and a continuous wave mode of irradiation. For ION recovery, the irradiation was performed on three areas between the upper lip philtrum, lateral nasal alae, lower eyelid, malar eminences and continuation of the lip commissure line (total of 10 points). For IAN recovery, the irradiation was performed near the mandibular canal, from the mandibular angle to the chin area (7 points) and from the mental foramen area to the midline (9 points). The design of the irradiation modality is shown in Figure 3.

The analysis was carried out using an electrodiagnostic test (blink reflex test) and a two-point discrimination test (TPD). The blink reflex test showed significant improvement in the function of ION and IAN, while the TPD described a positive effect of PBM on IAN.

## 4. Discussion

### 4.1. Photobiomodulation and Mitochondrial Bioenergetics

**Figure 4:**
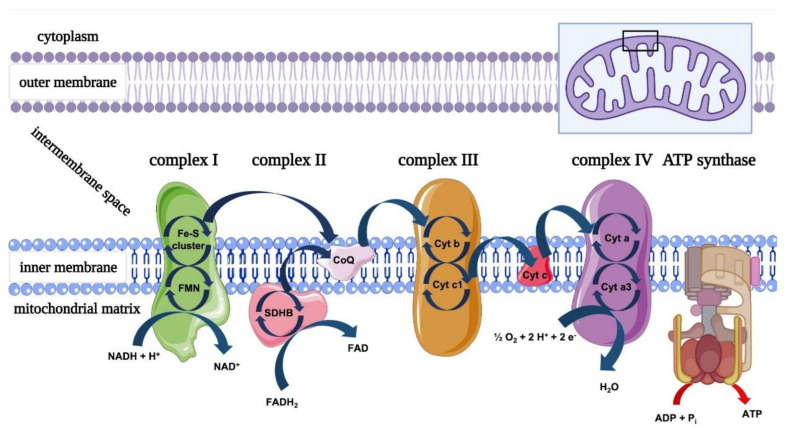
Organization of the oxidative phosphorylation (OxPhos) machinery. The respiratory chain, located in the inner mitochondrial membrane, consists of five multimeric protein complexes: reduced nicotinamide adenine dinucleotide (NADH) dehydrogenase–ubiquinone oxidoreductase (complex I) contains eight Fe-S clusters involved in the transfer of electrons from reduced flavin mononucleotide (FMNH2) to ubiquinone; succinate dehydrogenase–ubiquinone oxidoreductase (complex II) contains a heme b prosthetic group in its anchor domain, which is essential for the structural integrity and function of the complex; ubiquinone–cytochrome c oxidoreductase (complex III) contains a cytochrome b subunit with two heme moieties, a cytochrome c1 subunit with one heme and a Rieske protein subunit (UQCRFS1) with a [2Fe-2S] cluster, and finally, the cytochrome c oxidase (complex IV), which mediates the final step in the electron transport chain, by catalysing the reduction of oxygen to water. It contains two heme-a moieties and two Cu centres, all of which participate in the electron transfer process to the ATP synthase. Image created with BioRender.com.

The photochemical interaction between light and organisms has been described in photoautotrophic and photoheterotrophic life forms, which use these interactions to convert light energy into metabolic chemical energy through cellular respiration. In animals and humans, the ability of specific cells to respond to light stimuli is experienced by all; it underlies vitamin D production and visual processes [65,66,67]. This occurs thanks to specific molecules, photoreceptors, in specialised cells, which are involved in metabolic pathways directly related to light–cell interactions [67]. However, common, unspecialised and ubiquitous molecules called photoacceptors are also found in organisms. Despite the fact that their light-unrelated contribution to cell physiology is known, their role in the ability of light to modulate non-plant cell bioenergetic metabolism is not common knowledge. The features of these molecules, already empirically appreciated by the ancient civilisations through sun–light therapies, were scientifically described for the first time by Mester [68], around fifty years ago. Nowadays, despite descriptions of cellular targets for light such as latent transforming growth factor-beta (TGF-β1) [69], S-nitrosylated NO or transient receptor potential channels (TRPC) [70], the most characterised light–cellular interaction involves the mitochondria complexes [71] (Figure 4). This organelle may directly or indirectly play a key role in photobiomodulation.

Karu, Passarella and Pastore first described the ability of mitochondria to interact with red and NIR light [72,73]. They showed the primary step in cellular interactions with light in the 600 nm wavelength range is the absorption of photoenergy by the “metallic” photoacceptor located in the cytochrome of the mitochondrial respiratory chain complex-IV (cytochrome c oxidase (CoX)). The energised molecules may lead to increased synthesis of ATP and consequent modulation of ROS, Ca^2+^ and NO cellular concentrations. 

Photons, as bosons, can carry an electromagnetic force and behave as a power source. In this way, the photoacceptor can absorb photon energy and reach an electronically excited state, leading to a temporary change in both conformation and function. In this way, light, as reviewed in our previous paper, can play with the cell’s perspective [30,31,32]. 

Pastore et al. [73] showed increased oxygen consumption in mitochondria irradiated with 632 nm laser light, and Yu et al. [74] observed increased activity of mitochondrial complex-III and -IV when low energy light with a 660-nm wavelength was used. Conversely, higher energy irradiation using a handpiece with a Gaussian profile drastically increased the complex-I, -III and -IV activities, uncoupling the respiratory chain [74]. 

Previously, our team showed that, like the lower wavelengths and energies, 808 nm and higher energy irradiation with a handpiece with a flat-top profile can selectively stimulate complex-IV. At this wavelength, complex-III is also excited but poorly; complex-I and -II are not affected, and the mitochondrial coupling is maintained [75]. The same experiment in unicellular organisms confirmed the results above, showing balanced and increased oxygen consumption and ATP production [76,77]. Increasing the wavelength up to 980 nm maintains the targeting of complexes-IV and -III [78], which are strongly stimulated. At 1064 nm, the involvement of complex-I is seen for the first time, but complexes III and IV are also influenced, while the extrinsic mitochondrial membrane complex-II is again nonresponsive to photons [79]. 

As discussed in our previous papers [79], the data observed above cannot be explained by ordinary metal vs. light variation in the coefficient of absorption. The light-cytochrome model seems not to be able to explain the lack of effect on complex-II, which exhibits a possible heme photoreceptor like the others [80] and the effect on complex-III and -I only at some wavelengths and energies. The cytochrome “metal” (copper and iron) does not drastically change its coefficients of absorption as the wavelength increases up to 1064 nm [79]. In order to explain the entire process of PBM, a broader vision is likely to be necessary, taking into account another interactor such as water.

The interest in water is also stimulated by the evidence that, when moving from 415 nm to NIR, the absorbance of CoX decreases while the ATP production unexpectedly increases [81]. Irradiation with 415-nm light results in a drop in ATP concentration in adipocytes [82]. 

In the metabolic economy of the cell, organelles and molecules need to be synchronised for consistent cooperation. Organelles are surrounded by molecules such as proteins, glycoproteins, lipids and nucleic acids but also water, which is mostly bounded. Interestingly, bound water shows an almost crystalline structure, differing from free-water density. Its heat conductivity is much higher and the dielectricity-related frequencies much lower, and it has peculiar electron-conducting properties compared to its free form. Additionally, the bound water forms peculiar layers with Zeta potential and a characteristic bound water “exclusion zone” (EZ) for random water [83,84,85]. This characteristic of the bound water environment borders the hydrophilic side of the phospholipid bilayer membrane of organelles and their compartments. 

Recent evidence by Pollak et al. [86,87] could support advancement in the understanding of PBM. Indeed, the electromagnetic energy of the photon, especially in NIR and infrared light, is able to separate water molecules and, therefore, by building order and separating charge, recharge the EZ [83,84,85]. Sommer [88] proposed a model involving nanoscopic interfacial water layers surrounding biological membranes and biomolecules, which seem to interact with infrared light, reducing the water viscosity.

NIR and infrared light have also shown the ability to affect vibrations of water’s ionic species, and polychromatic light (750–2000 nm) is able to perturb the energy of hydrogen bonds [89]; alterations of membrane fluidity and the formation of H3O^+^ and OH^−^ were then observed. 

Hence, light is able to interact with water, reducing its viscosity and leading to a possible increase in the rotation speed of ATP synthase [88] and modifying the cellular membrane fluidity. This may lead to changes in the conformation and interconnection among the mitochondrial crests and/or cardiolipin, with consequent influence on mitochondrial respiratory chain activity [78,90]. Plus, the change in the water dissociation products could also affect the bioenergetic behaviour of mitochondria, despite the charge transfer process in the aqueous solution might not play a pivotal role in the mitochondria electron and proton [91]. The “water affaire” seems, therefore, to be of interest for the future of PBM, because water as a photoacceptor can influence the mitochondrial metabolic activities, likely in a similar way to other cellular pathways. 

Lastly, lipids in the near-infrared wavelength region showed two peaks of absorption at 1210 and 1720 nm, but also, a subsequent third peak in the 900–1000-nm range [92]; as previously described, this could lead to a membrane fluidity modification and an influence on mitochondria. The change in membrane fluidity and the vibrational modification of water molecules can play a role in Ca^2+^ release from the TRPC [93] but, also, in a mitochondrial reserve [94,95]. In a previous review, we showed that the effect of light on Ca^2+^ homeostasis can have a key role in mitochondrial metabolism and NO release, in a peculiar system that could be described as “like a dog chasing its own tail” [71].

Since photobiomodulation has been theoretically described and evidenced, the prediction and standardisation of the results need attention. As extensively described by de Freitas and Hamblin [70] in their review of proposed mechanisms for photobiomodulation, “there is a biphasic dose-response curve, or hormesis, in which too low or too high doses (fluence (J/cm^2^), irradiance (mW/cm^2^), delivery time or a number of repetitions) can lead to no significant effect or, sometimes, inhibitory effects”. Additionally, a recent study [78] pointed out that hormetic behaviour can certainly explain the performance of PBM, but the effect on mitochondria seems to occur in narrow windows of positive effect/no effect/negative effect, more than a watershed upper at or lower of. Therefore, the statement that low energies and powers cure while higher ones harm must be reconsidered, while preventing thermal effects [78].

### 4.2. In Vivo Preclinical Studies

Regarding nerve regeneration, as suggested by Diker et al. [36], most of the original studies involve animal sciatic nerves, and only a small number investigated the effects of PBM following the injury of the trigeminal nerve branches. Despite not being included in the review process due to nonadherence to many points of the ARRIVE guidelines, nine comparative research articles [34,35,38,40,42,43,44,45,46] using a total of 273 rats showed the effects of PBM at different wavelengths and phototherapy parameters on the regeneration of trigeminal nerve branches, neurosensory recovery and pain. These effects are most likely the consequence of cell physiology modulation, resulting in a decrease in substance P (SP), vanilloid transient potential receptor of subtype-1 (TRPV1), peptide related to the calcitonin gene [34], matrix metalloproteinases [35], laminin, myelin protein zero, neurofilaments [38] and brain-derived neurotrophic factor [45], and an increase in the nerve growth factor level [45]. These proteins are involved in the inflammation, pain and regeneration outcomes after nerve injury [96,97,98]. This evidence supports the consistent results of other authors [36,37,39,41], who have demonstrated the effectiveness of PBM in nerve regeneration and neurosensory recovery. In fact, inferior alveolar nerve crush injury recovered faster than control when PBM therapy was used in rats [36,37] and rabbits [39] models, and the same behaviour was observed for the injured mental nerve of rats [41].

Additionally, the wavelengths of 808 and 830 nm showed a better effect than the 660 [36] and 980-nm wavelengths [37]; 660-nm light did not improve the axon myelinisation and regeneration, while the 980-nm light influenced the neurosensory recovery, but more weakly.

The most well-supported reason behind this lies in the different penetration depths of different wavelengths, due to their coefficients of absorption with melanin and water. Indeed, melanin absorption is still evident in the red-light range of 600–700 nm, as well as a water absorption increase from 800 to 1000 nm. The 800-nm spectral region, therefore, shows greater transmittance across the skin thickness. This is of relevance to IAN because of its anatomic position, as radiation has to pass through the mandibular bone before reaching IAN injuries [99]. Additionally, the effect was correlated with a decrease in inflammation and pro-degenerative cellular pathway activity, as demonstrated by the decrease in nuclear factor-kB (NF-kB), tumour necrosis factor-alfa (TNF-a) and interleukin-1beta (IL-1b) [37]. 

As previously shown in Section 1.2, mitochondria have an important role in preventing or reversing dysfunction in the course of peripheral nervous system damage and peripheral neuropathy, and PBM may influence this role.

Additionally, from a cellular point of view, PBM could probably act on the Schwann cell, which plays a key role in the regeneration of the axon [100]. Evidence, suggest that Schwann cells may be used therapeutically to facilitate axon regeneration after trauma [101]. Interestingly, Schwann cell mitochondria are regulators in the development, maintenance and regeneration of peripheral nerve axons [102].

Indeed, PBM immediately increases ATP production in the ototoxicity neurodegeneration model, and it is quickly consumed to counteract neurodegeneration [103]. Additionally, PBM’s preservation of mitochondrial dynamics and functions can recover apoptotic neuronal death in global cerebral ischemia [104]. The increase in ATP levels following PBM can therefore improve the regeneration of the injured nerve and counteract its degeneration. This also occurs by the inhibition of NO production and ROS, as well as modulation of Ca^2+^ homeostasis to protect neurons from toxic and proinflammatory effects, as described in Section 1.2; this mechanism was also demonstrated in endothelial dysfunction treated with PBM [31]. The possible role of mitochondria in nerve regeneration recovery through PBM therapy is also supported by recent evidence on nervous cells. Authors showed the increase of neurons stem cell differentiation through modulation of cellular metabolism and redox status [105], of the activity of respiratory chain complexes in an apparent dose- and time-dependent manner [106], of modulation of mitochondrial dynamics through mitofusin 2 and dynamin-related protein 1 expression [107], as well as the key role of cytochrome c oxidase [108], cell metabolism, oxidative stress, mitochondrial membrane potential and calcium flow in neuroprotection of sensory neurons [109].

Additionally, consistent results showed that persistent pain states are mediated by cooperative actions of TRPV1, glutamate, SP and calcitonin gene-related peptide (CGRP). However, Martins et al. [38] observed that a decrease in TRPV1 and an increase in glutamatergic transmission induced by PBM with 904-nm light is an accredited way to explain the reduction of neuropathic pain related to the trigeminal nerve. Calcium may have also a role in this issue. Indeed, de Freitas Rodrigues [34] and Pigatto et al. [110] showed that PBM decreased TRPV1 expression, and Amaroli et al. [111] demonstrated that 808-nm laser light induced calcium-dependent glutamate release from nerve terminals, only in the presence of mitochondria. It is known that glutamate release is associated with ATP [112]. Plus, glutamate may be beneficial in nerve injury due to the upregulation of *N*-methyl-d-aspartate receptors (NMDARs), which promotes the migration and survival of nerve cells in vitro and in vivo [113]. Therefore, the connection between light and mitochondria occurring in PBM can also influence many cellular pathways involved in damaged nerve recovery. 

Undoubtedly, the mechanism by which PBM can directly or indirectly support injured nerve recovery via mitochondrial bioenergetics has been shown and appears to be plausible. Nevertheless, the literature shows contradictory results about the effects and optimum wavelength of PBM [30,31]. Our review process using the ARRIVE guidelines identified the work of Diker et al. [3] as the most consistent of the preclinical papers selected. The authors induced “IAN injury by a clamp, for thirty seconds with micro-forceps, at the level of the 2-mm rostral to the mandibular foramen, where the main trunk of IAN divided into two large branches”, and at the 30th postoperative day, histopathological and histomorphometric analyses were performed. The 808-nm PBM group presented the most axon fibres within the optimal g-ratio range (0.55–0.69), the number of axons (unit square micrometre) was significantly higher and revealed a lower level of degeneration in the myelinated nerve fibres. Compared to the injured but not irradiated control group and the 660-nm PBM group, the tissue sections of the 808-nm PBM group revealed preserved general structure and lower oedema and collagen fibre deposition in the intercellular connective tissue.

The authors concluded that a therapy based on laser parameters, such as: frequency 50 Hz of a pulsed wave, peak radiant power of 100 mW, average radiant power of 70 mW, the irradiance at the target of 2.5 W/cm^2^, radiant exposure of 100 J/cm^2^, radiant energy of 2.7 J/session, total radiant energy 27 J, exposure duration of 80 s, irradiation at only one point of 0.028 cm^2^ beam spot size, in noncontact mode, with 10 treatments in total, delivered once every 3 days over 30 days, constituted aid for IAN injury recovery in a preclinical rat model, and could be a promising method to support clinical applications.

### 4.3. Clinical Studies

Therefore, despite the effectiveness and mechanistic and functional behaviours of PBM having been described in mitochondria and animal models, the switching to human patients is not easy, due to the features of the different wavelengths, which may lead to unpredictable penetration through tissues, according to their type, thickness and physiology. 

However, thanks to not-designed and observational studies, various authors have described the effectiveness of PBM for the treatment of IAN [53,54,55,56,57,58,59], lingual [53,59] and maxillary [53] nerves, injured by third molar and odontoma removal, osteotomy, dental implant placement and facial trauma. These results were supported by up to 2 years of follow-up [54,56,57,58].

Concerning the RCT studies, only one was assessed to have a low risk of bias. Taken together, the results support our previous statement that pointed out the necessity to support the research on PBM with more careful experimental set-ups and description [30,31]. In fact, these RCT studies that raise some concerns regarding bias failed to blind the operator performing the irradiation. However, the patients were blinded, as was the operator that collected and analysed the data. As described in the results, the use of scales of valuation can involve arbitrary judgment but is considered an accredited tool for many types of research on nerve regeneration.

Therefore, the results of the RCT studies selected [47,48,49] may be reasonably reliable and confirm the utility of PBM in regeneration and neurosensory recovery, with the exception of a study [50] employing PBM at 830 nm; 0.4 W; 2.67 W/cm^2^; 20 J/cm^2^, 40 J/cm^2^; 6 J intraorally, 3 J extra-orally; 7.5 s, 15 s; 0.15 cm^2^; continuous wave, which did not show any effect. A dangerous effect was not, however, observed at the 3-month follow-up.

Lastly, support for clinicians can be derived from the double-blind split-mouth study of Haghighat et al. [47], where PBM was used at 800 nm; 0.2 W; 0.2 W/cm^2^; 12 J/cm^2^; 12 J; 60 s per point; 1 cm^2^; continuous wave mode of irradiation.

## 5. Conclusions

Concerning the questions of our review, we can conclude the following:

1. The reliability of photobiomodulatory event strongly bases on biological and physical–chemical evidence. Its principal player is the mitochondrion, whether its cytochromes are directly involved as photoacceptor or indirectly through a vibrational and energetic variation of bound water and water as a photoacceptor.
2. Moving from a microscopic point of view to preclinical and clinical, photobiomodulation seems to confirm its role as effective medical support for trigeminal disease. Irradiations in both pulsed and continuous wave mode affect the IAN nerve regeneration and neurosensory recovery through accurate wavelengths and doses.
3. The 808-nm and 100 J/cm^2^ (0.07 W; 2.5 W/cm^2^; pulsed 50 Hz; 27 J per point; 80 s) on rats and 800-nm and 0.2 W/cm^2^ (0.2 W; 12 J/cm^2^; 12 J per point; 60 s, CW) on humans irradiated with the modalities previously described, resulted as trustworthy therapies, which could be supported by extensive studies.

## Figures and Tables

**Table 1:** Included studies. Preclinical in vivo studies on photobiomodulation and trigeminal branches nerves recovery, selected after inclusion and exclusion criteria and ARRIVE guideline screening. The table shows the schematic design of the experimental set-up on animals and the results.

Study	Animal/Number	Parameters	Therapy	Area/Damage	Method	Results
[36] *	Rat/36 (3 groups)	660–808 nm; 0.07 W; 100 J/cm^2^; 2.5 W/cm^2^; pulsed 50 Hz; 27 J per point; time = 80 s; distance = n.s.; spot area = 0.028 cm^2^	Start = immediately End = 30 days after 1 point every 3 days	Mandibular canal/inferior alveolar nerve crush injury (surgery)	Nerve regeneration by histomorphological analysis	808 nm: increment of axons and myelinization 660 nm: no effect
[37]	Rat/72 (4 groups)	810–980 nm; 0.2 W; 6 J/cm^2^; 0.4 W/cm^2^; continuous wave; 3 J per point; time = 15 s; distance; contact; sport area = 0.5 cm^2^	Start = a day after surgery End = 30 days after 3 points daily	Mandibular canal/inferior alveolar nerve crush injury (surgery)	Neurosensory recovery: von Frey test Immunoblot: BDNF; NF-kB; TNF-a; IL-1 b	Neurosensory recovery is improved by both wavelengths. NF-kB; TNF-a; IL-1b decreased by both wavelengths. 810 nm better than 980 nm
[41]	Rat/48 (4 groups)	808 nm; 0.1 W; 120 J/cm^2^; 3.57 W/cm^2^; continuous wave; time = 22–33 s; spot area = 0.028 cm^2^; 3.5 J per point	Start = a day after surgery End = 20 days after 1 point daily	Mental nerve/nerve crush with ultrafine forceps	Nerve regeneration: TEM	Increase of regeneration after 14 days up to 20 days of irradiations
[39]	Rabbit/5 (split mouths)	830 nm; 6.0 J/cm^2^; 0.06 W/cm^2^; continuous wave; time = 90 s	Start = immediately End = 10 days after 4 points daily	Mandibular canal/inferior alveolar nerve crush injury (surgery and implant into Gore-Tex tube)	Nerve regeneration by histomorphological analysis	Nerve regeneration in samples with both only tube and tube + PBM. PBM improves the effect

The symbol * indicates the paper that obtained the higher score after the ARRIVE screening. BDNF = brain-derived neurotrophic factor; IL-1b = interleukin-1beta; NF-kB = nuclear-factor-kB; PBM = photobiomodulation; TNF-a = tumour necrosis factor-alpha.

**Table 2:** Included randomised control trial (RCT) studies. Clinical studies on photobiomodulation and trigeminal branches nerves recovery selected after the inclusion and exclusion criteria and PRISMA, COCHRANE guideline screening.

Study	Study/Patients/Age	Parameters	Therapy	Damaged Area/Cause	Method	Results
[47] *	RCT, double-blinded/12/average age 23.4	810 nm; 0.2 W; 0.2 W/cm^2^; 12 J/cm^2^; 12 J; time = 60 s per point; 1 cm^2^; continuous wave	Start = immediately End = 14 days after 16 points on maxilla and 10 points on mandible side. Irradiation 1, 5, 10 and 14 days after surgery	Inferior alveolar nerve and infraorbital nerve/Bilateral sagittal split osteotomy, Le Fort 1 osteotomy	2-point discrimination test Blink-test (3-months follow-up)	Improve of neurosensory recovery
[48]	RCT, double-blind/40/average age 26	810 nm; 0.07 W; 0.14 W/cm^2^; 8.4 J/cm^2^; 4.2 J per point; 60 s; 0.5 cm^2^; continuous wave	Start = immediately End = 2 weeks 1 intraoral point and 3 extraoral points 1, 2 and 3 days after surgery and every other day for the next two weeks (10 section in total)	Inferior alveolar nerve/sagittal split osteotomy	2-point discrimination test Thermal test Contact direction test Pinprick test (12-months follow-up)	Rapid progression of the nerve healing process
[49]	RCT, double-blinded/33/average age 22	810 nm; 0.1 W; 0.356 W/cm^2^; 32 J/cm^2^; 9 J per point; 90 s; 0.28 cm^2^; continuous wave	Start = a day after surgery End = after 28 days 3 intraorally points at session. Irradiation at day 2, 3, 5, 10, 14, 21 and 28 after surgery	Inferior alveolar nerve/Bilateral sagittal split osteotomy	VAS 2-point discrimination test Thermal test Pain discrimination (6-months follow-up)	Recovery of neurosensory Impairment of mandibular nerve
[50]	RCT/35/average age 39.97	830 nm; 0.4 W; 2.67 W/cm^2^; 20 J/cm^2^, 40 J/cm^2^; 6 J intraorally, 3 J extraorally; 7.5 s, 15 s; 0.15 cm^2^; continuous wave	Start = immediately End = 20 sessions after From 1 to 8 points	inferior alveolar nerve and lingual nerve/local anesthetic, third molar odontectomy, and dental implant placement	visual analogue scale clinical neurosensory testing (3-months follow-up)	No effect
[51]	RCT, doubled-blind, split osteotomy/20/average age 35	780 nm; 0.07 W; 1.75 W/cm^2^; 157.5 J/cm^2^; 6.3 J per point; 90 s; 0.04 cm^2^, continuous wave	Start = variable End = after 5 sessions with intervals of three to four weeks between the sessions	Inferior alveolar nerve/Bilateral sagittal split osteotomy	Semmes-Weinstein monofilament test (12-months follow-up)	improve neurosensory recovery
[52]	RCT, Triple-blind, split osteotomy/20/average age 23	980 nm; 0.1 W; 0.2 W/cm^2^; 12 J/cm^2^; 6 J per point; 60 s; 0.5 cm^2^; continuous wave	Start = a day before osteotomy End = 28 days after 12 points per session 1, 3, 7, 14, 21 and 28 day postoperatively	Inferior alveolar nerve/Bilateral sagittal split osteotomy	2-point discrimination test Thermal test Contact direction test (1-month follow-up)	improve neurosensory recovery

The table shows the schematic design of the experimental set-up on patients and the results. The symbol * indicates the paper that obtained the higher score after the Cochrane screening.

**Table 3:** No RCT clinical studies on photobiomodulation and trigeminal branches nerves recovery, selected after inclusion and exclusion criteria. The table shows the schematic design of the experimental set-up on patients and the results.

Study	Study/Patients	Parameters	Therapy	Damaged Area/Cause	Method	Results
[53]	Clinical-cases/ 125 patients	808 nm; 0.1 W; 3.57 W/cm^2^; 100 J/cm^2^; 2.8 J per point; 28 s; 0.028 cm^2^; continuous wave	Start = variable End = at the average number 13 laser sections. 1–2 section per week	Inferior alveolar, mental, lingual and maxillary nerves/orthognathic surgery, Inferior alveolar nerve lateralization, third molar extraction, dental implant placement, facial trauma	visual analogue scale (VAS) (at the end of sessions)	Better recovery of sensitivity in younger (14–25 y/o) than older (>61 y/o) patients. PBM acts better in orthognathic surgery and facial trauma than in other cases.
[54]	Case report/1	660 nm and 808 nm; 0.1 W; 3.57 W/cm^2^; 140 J/cm^2^; 4 J per point; 40 s; 0.028 cm^2^; continuous wave	Start = a day after surgery End = after 10 sessions First irradiation with 660 nm followed by 808 nm. 52 points irradiated per session	Inferior alveolar nerve/removal odontoma	VAS (2-year follow-up)	Improvement of neurosensory
[55]	Case series/20	808 nm; 0.1 W; 3.57 W/cm^2^; 100 J/cm^2^; 2.8 J per point; 28 s; 0.028 cm^2^; continuous wave	Start = two day after surgery End = after 10 sessions 25 points at session. Irradiation every 72 h	Inferior alveolar nerve/Bilateral sagittal split osteotomy	VAS (at the end of sessions)	Improvement of neurosensory
[56]	Case series/42	810 nm; 0.1 W; 0.353 W/cm^2^; 31.8 J/cm^2^; 9 J per point; 90 s; 0.283 cm^2^, continuous wave	Start = immediately End = 28 days after surgery Three intraoral applications on days 1, 2, 3, 5, 10, 14, 21, and 28 after surgery	Inferior alveolar nerve/Bilateral sagittal split osteotomy	VAS 2-point discrimination test Thermal test (2-years follow-up)	Improve neurosensory recovery
[57]	Case series/13	820 nm; 0.07 W; 0.55 W/cm^2^; 46 J/cm^2^; 6 J per point; 86 s; 0.13 cm^2^; continuous wave	Start = immediately End = from 20 to 63 days after (mean, 31 days). 4 points per treatment along with the distribution of the inferior alveolar nerve, for a total of 20 treatment	Inferior alveolar nerve/Bilateral sagittal split osteotomy	Semmes Weinstein monofilaments test, Thermotester, VAS (at the end of sessions)	Improve neurosensory recovery
[58]	Prospective study/6	820–830 nm; 0.55 W; 4.2 W/cm^2^; 46 J/cm^2^; 6 J per point; 86 s; 0.13 cm^2^	Start = immediately End = 7 days 4 points per treatment along with the distribution of the inferior alveolar nerve, irradiated at the day 2, 3, 4 and 7 after surgery	Inferior alveolar nerve/Bilateral sagittal split osteotomy	VAS 2-point discrimination test Thermal test Contact direction test Pinprick test (2-years follow-up)	Improve neurosensory recovery
[59]	Case report/4	820–830 nm; 0.05 W; 0.25 W/cm^2^; 22.5 J/cm^2^; 4.5 J per point; 90 s; 0.2 cm^2^	Start = immediately End = 5 weeks after 5 point for session 3 times per week	inferior alveolar nerve or the lingual nerve/third molar odontectomy	brush stroke directional discrimination test, 2-point discrimination test, VAS (9-months follow-up)	Recovery of neurosensory Impairment of nerves
[60]	Case series/57	904–910 nm; frequency of 1 to 80 kHz; pulsed and superpulsed emissions (200 ns of pulse duration); peak power 40 W; 0.008 to 0.5 W of average power	Start = variable End = after 10 sessions, carried out weekly	inferior alveolar nerve/oral surgical injury	2-point discrimination test Thermal test Contact direction test Pinprick test (10-weeks follow-up)	neurosensory recovery in 83% of patients
[61]	Case series/20	808 nm; 0.05 W; 0.016 W/cm^2^; 3 J/cm^2^; 9.42 J per point; 188 s; 3.14 cm^2^; continuous wave	Start = variable End = after 7 sessions once every two days 1 point	inferior alveolar nerve/third molar odontectomy	VAS Clinical neurosensory test (at the end of sessions)	improve neurosensory recovery
[62]	Case series/11	808 nm; 0.017 W; 0.2 W/cm^2^; 4 J/cm^2^; 44 J per session; 20 s; = 0.088 cm^2^; continuous wave	Start = variable End = after 15 session 15 points intra and extraorally twice per week	inferior alveolar nerve/third molar odontectomy	Zuniga–Essick score, British Medical Research Council scale, VAS (at the end of sessions)	improve neurosensory recovery

## Data Availability

Data available on request from the authors.

## Supplementary Materials

The following are available online at https://www.mdpi.com/article/10.3390/ijms22094347/s1, Figure S1: Flow chart demonstrating the selection process by PRISMA guideline, Figure S2: Hierarchy of the clinical studies, Table S1: ARRIVE Essential 10 guideline, Table S2: Excluded preclinical in vivo studies, Table S3: Risk-of-bias tool for randomized trials by Cochrane RoB 2.
