# Supplementary material for: Mitochondrial Bioenergetic, Photobiomodulation and Trigeminal Branches Nerve Damage, What’s the Connection? A Review
Source: Int J Mol Sci. 2021 Apr 21;22(9):4347. doi: 10.3390/ijms22094347 (PMC8122620; doi:10.3390/ijms22094347)
Supplement: Supplementary file 1 [file ijms-22-04347-s001.zip › ijms-1187809-supplementary.pdf]

International Journal of
Molecular Sciences

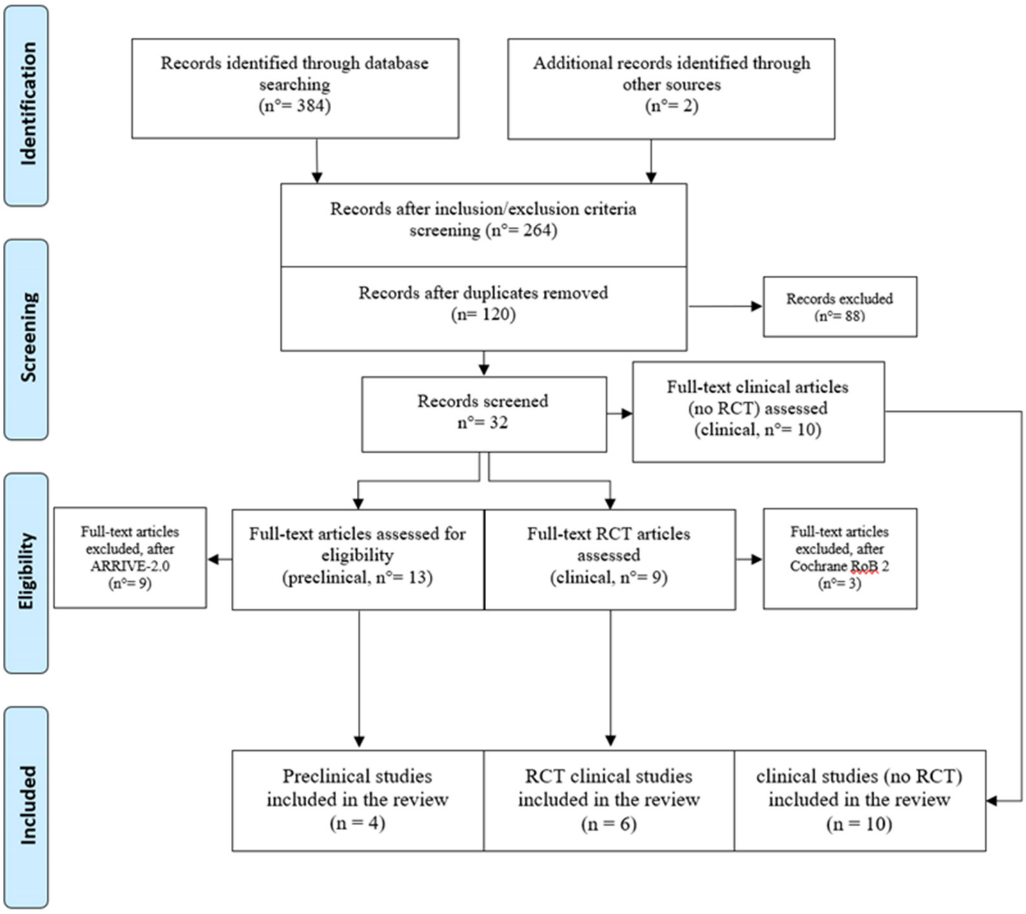

Figure S1. Flow chart demonstrating the selection process by PRISMA guideline.

Table S1. ARRIVE Essential 10 guideline. These items represent the basic data to include in a manuscript on animals' research. Without this information, readers and reviewers cannot assess the reliability of the findings. In Green the article with high score and considered sufficiently reliable. In Yellow the articles that suggest some concerns on their reliability. In Red the article excluded. Y = yes, PY = probably yes; N = no; PN = probably no.

Article	Study design	Sample size	Inclusion and exclusion criteria	Randomisation	Blinding	Outcome measures	Statistical methods	Experimental animals	Experimental procedures	Results
34	Y	Y	N	N	N	Y	Y	Y	Y	Y
35	PY	PN	N	N	N	Y	Y	Y	Y	Y
36	Y	Y	N	Y	Y	Y	Y	Y	Y	Y
37	Y	PN	N	Y	Y	Y	Y	Y	Y	Y
38	Y	PN	N	N	Y	Y	Y	Y	Y	Y
39	Y	PN	N	Y	Y	Y	Y	Y	Y	Y
40	Y	PN	N	Y	N	Y	Y	Y	Y	Y
41	Y	PN	N	Y	Y	Y	Y	Y	Y	Y
42	Y	PN	N	N	N	Y	Y	Y	Y	Y
43	Y	PN	N	Y	N	Y	Y	Y	Y	Y
44	Y	PN	N	Y	N	Y	Y	Y	Y	Y
45	Y	PN	N	N	N	Y	Y	Y	Y	Y
46	Y	PN	N	Y	N	Y	Y	Y	Y	Y

Table S2. Excluded studies. Preclinical in vivo studies on photobiomodulation and trigeminal branches nerves recovery, selected after inclusion and exclusion criteria and ARRIVE-guideline screening. The table shows the schematic design of the experimental set-up on animals and the results. BDNF = brain-derived neutrophic factor; CGRP = calcitonin gene-related peptide; CFA = complete Freund's adjuvant; MMP = matrix metalloproteinase; MPZ = myelin protein zero; NFs = neurofilaments; NGF = nerve growth factor; OXT = oxytocin necrophysin precursor; PBM = photobiomodulation; SP = substance P; TRPV-1 = vanilloid transient potential receptor; TGF = tumor growth factor; TEM = transmission electron microscopy.

Study	Animal/ Number	Parameters	Therapy	Area /Damage	Method	Results
34	Rat/40 (4 groups)	904nm; 0.043 W; 5.95 J/cm^2; 0.3338 W/cm^2; pulsed 9500 Hz; 60 ns; 0.7J per point; time = 18 sec per point; distance = 1cm; spot area = 0.13 cm^2	Start: 2 days after surgery End = 20 days after 10 points daily	Trigeminal nucleus/ tempo-mandibular joint disc injury (surgery)	Neurosensory recovery: von Frey test Immunoblot: SP, TRPV-1; CGRP	Reduction of pain through two therapy section up to 20. Decrement of SP, TRPV-1; CGRP
35	Rat/40 (4 groups)	780nm; 0.04 W; 20 J/cm2; continuous wave; 08J per point; time = 20 sec; distance = contact; spot area = 0.04 cm2	Start: immediately End = 9 days after At the 0, 3, 5, 7, 9 day	Trigeminal nucleus/ tempo-mandibular joint inflammation (injection with CFA)	Evaluation of: hyperalgesias and allodynis. Gelatin zymography: MMP, TGF	Decrements of inflammation Decrement of MMP
38	Rat/40 (4 groups)	904nm; 0.07 Wpk; 6 J/cm2; pulsed 9500 Hz; 60 ns; spot area = 0.1 cm2	Start: 2 days after surgery End = 10 days after 1 point daily	Mandibular canal/ inferior alveolar nerve crush injury (surgery)	Nerve regeneration by TEM Immunoblot: laminin; MPZ; NFs;	Increment of inferior alveolar nerve regeneration Decrement of laminin, MPZ, NFs
40	Rat/30 (3 groups)	810nm; 0.1W; continuous wave; time = 500 seconds	Start = immediately End = 2 days after 1 point every other day	Infraorbital nerve/binding by 6-0 silk	Neurosensory recovery: electrical stimulation of dental pulp	Cortical excitation suppressed by OXT and PBM together. Only OXT or PBM had not effect
42	Rat/27 (3 groups)	618 nm; 6 J/cm2; 0.02 W/cm2; time = 300 sec; distance = contact	Start = immediately End = 21 days after 1 point daily	Mental nerve/nerve suturated	Nerve regeneration by histomorphological analysis	Increase of regeneration by ozone and PBM together.
43	Rat/42 (5 groups)	830nm, 0.03W; 6 J/cm2; 0.256 W/cm2; continuous wave; time = 24 sec; distance = contact; spot area = 0.116 cm2	Start = a day after surgery End = 5 week after 1 point 3 times weekly	Buccal branch of facial nerve/transected by surgery	Nerve regeneration by TEM	Increase of axon regeneration
44	Rat/42 (5 groups)	830nm; 0.03W; 6.2 J/cm2; 0.2586 W/cm2; continuous wave; 2.16J per point; time = 24 sec; distance = contact; spot area = 0.116 cm2	Start = a day after surgery End = 5 week after 1 point 3 times weekly	Facial nerve/transected by surgery	Nerve regeneration by TEM	Increase of axon regeneration and function

45	Rat/24 (5 groups)	904nm; W; 6 J/cm2; W/cm2; pulsed 9500 Hz; 60ns; 6J/cm2; 6J per point; time = 18 sec; distance = no-contact; spot area = 0.5 cm2	Start = two day after surgery End = 20 days after 1 point every 2 days for 10 sessions	Inferior alveolar nerve/Crile hemostatic clamp	Nerve regeneration by sensory test Immunoblot: NGF, BDNF	Increase of axon regeneration Immunoblot = NGF level increases, BDNF level decreases
46	Rat/30 (3 groups)	850nm; 0.1W/cm2; 1 or 3 MHz or continuous wave; 4J; time = 32 sec;	Start = immediately End = 21 days after 1 point daily	Left facial nerve/surgical clamp	Nerve regeneration by histomorphological evaluation after PBM or ozone therapy	Ozone therapy and PBM improve nerve regeneration. Ozone therapy is better than PBM

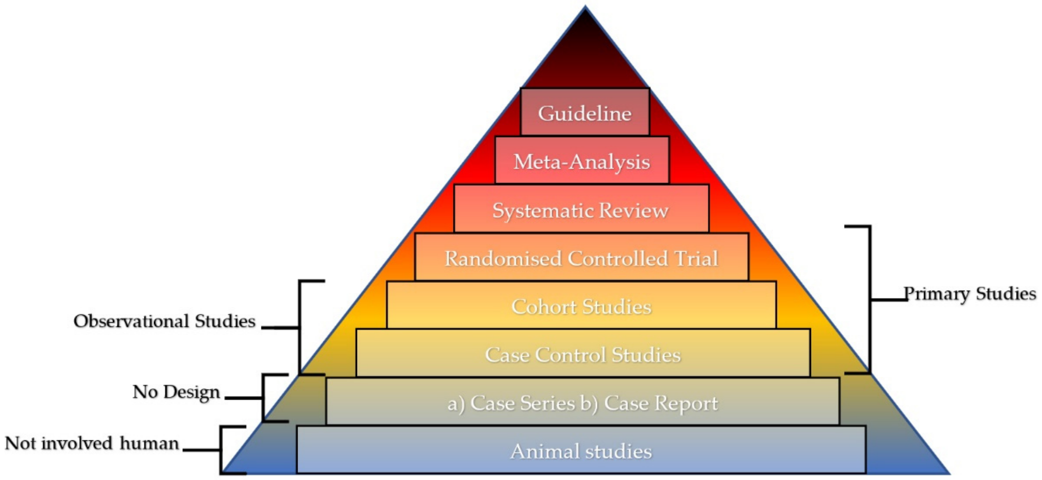

Figure S2. hierarchy of the clinical studies.

Table S3. Risk-of-bias tool for randomized trials by Cochrane RoB 2

STUDY	A	B	C	D	E	F	G	Risk of bias	Conflict of interest
63	NI	NI	PN	**Y**	PN	**Y**	PN	High risk	NI
48	PY	NI	PN	**Y**	**Y**	**Y**	PN	Some concerns	NONE
49	**Y**	**Y**	PN	**Y**	**Y**	**Y**	PN	Some concerns	NONE
64	NI	NI	NI	**Y**	PN	**Y**	PN	High risk	NONE
47	**Y**	NI	Y	**Y**	**Y**	**Y**	**Y**	Low Risk	NONE
50	**Y**	**Y**	PN	NI	**Y**	**Y**	PN	Some concerns	NI
51	NI	NI	PN	**Y**	**Y**	**Y**	PN	Some concerns	NONE
52	**Y**	NI	PN	NI	**Y**	**Y**	PN	Some concerns	NONE

Selection bias: (A) Random sequence generation; (B) Allocation concealment; (C) Blinding of participants and personnel; (D) Blinding of outcome assessment; (E) Complete outcome data; (F) Selective reporting; (G) other bias.
